# Frequency of nodular goiter and autoimmune thyroid disease and association of these disorders with insulin resistance in polycystic ovary syndrome

**DOI:** 10.4274/jtgga.2016.0217

**Published:** 2017-06-01

**Authors:** Melia Karaköse, Sema Hepsen, Erman Çakal, Müyesser Saykı Arslan, Esra Tutal, Şafak Akın, İlknur Ünsal, Mustafa Özbek

**Affiliations:** 1 Department of Endocrinology and Metabolism, Dışkapı Training and Research Hospital, Ankara, Turkey; 2 Department of Internal Medicine, Dışkapı Training and Research Hospital, Ankara, Turkey

**Keywords:** Polycystic ovary syndrome, autoimmune thyroid disease, Nodular goiter, insulin resistance

## Abstract

**Objective:**

Polycystic ovary syndrome (PCOS) is a frequent endocrine disease in women. Nodular goiter and autoimmune thyroid disease (AITD) are endocrinologic abnormalities that have high prevalence. The purpose of our study was to detect the prevalence of AITD and nodular goiter in patients with PCOS and investigate whether PCOS-related hormones and metabolic factors affect these thyroid disorders.

**Material and Methods:**

Ninety-seven women with PCOS and 71 healthy female volunteers were recruited into the study. Serum-free thyroxine, thyroid-stimulating hormone, anti-thyroperoxidase antibody and anti-thyroglobulin antibody levels were evaluated. Thyroid volume (TV) was calculated using ultrasonography.

**Results:**

The body mass index (BMI), Waist/Hip ratio, homeostasis model assessment insulin resistance (HOMA-IR), fasting blood glucose, triglyceride and low-density lipoproteins, and fasting insulin were significantly higher in the PCOS group (p<.05). The control group had significantly higher serum high density lipoprotein cholesterol results (p=.005). The mean TV was 11.4±4.7 mL in the PCOS group and 9.9±2.8 mL in the controls (p=.022). Twenty-nine patients with PCOS (29/97; 29.9%) had thyroid nodules, whereas only eleven control subjects had thyroid nodules (11/71; 15.5%) (p=.043). The frequency of AITD was significantly higher in the PCOS group (p=.001). A statistically significant relationship was found between TV and age, fasting glucose, HOMA-IR, BMI, and fasting insulin (p<.05). Participants with thyroid nodules were older and had higher fasting glucose, BMI, fasting insulin, and HOMA-IR values compared with those without thyroid nodules (p<.05).

**Conclusion:**

We demonstrated that TV and frequency of nodular goiter were increased in patients with PCOS. This result was related with insulin resistance. Therefore, we recommend that patients with PCOS must be investigated for the development of nodular goiter and AITD.

## INTRODUCTION

Polycystic ovary syndrome (PCOS) is a frequent endocrine disease with a prevalence of 5-10% in women at reproductive age (1). Hyperandrogenism, menstrual irregularities, infertility, and obesity are main features of this syndrome. Patients with PCOS have increased risk of metabolic syndrome, cardiovascular disease, insulin resistance, type 2 diabetes mellitus (type 2 DM), and endometrial carcinoma (2, 3, 4, 5, 6).

Nodular goiter and autoimmune thyroid disease (AITD) are both frequently seen disorders in endocrine practice (7). Autopsy and ultrasonography (USG) series showed that thyroid nodules had a high prevalence (19-50%) in the general population (8, 9). In women, the most common cause of hypothyroidism is AITD, which affects 5-20% of the young female population. The interaction of genetic and hormonal factors are important in the etiology of AITD (10, 11).

Although iodine deficiency, female sex and age are well-known risk factors for nodular goiter, the exact pathogenesis remains to be illuminated. The potential relation between insulin resistance and thyroid nodules was reported by Rezzonico et al. (12, 13), and insulin resistance was also accepted as a risk factor for cancer development. Patients with PCOS have increased risk of insulin resistance. The purpose of this study was to determine the prevalence of AITD and nodular goiter in patients with PCOS and investigate whether there was an effect of PCOS-related hormones and metabolic factors on these thyroid disorders.

## MATERIAL AND METHODS

Ninety-seven patients who were diagnosed as having PCOS between August 2015 and September 2016 in our hospital at the Department of Endocrinology and Metabolism outpatient clinic in accordance with the 2003 Rotterdam criteria were recruited to the study (14). Seventy-one age-matched healthy female volunteers were recruited to the study as a control group. The exclusion criteria were chronic systemic disease, and using drugs that could affect insulin sensitivity and lipid parameters. The study protocol was approved by the Ethics Department and all participants gave informed consent.

Anthropometric measurement, physical examination, and biochemical screening were made in all patients and controls. We obtained fasting blood samples from all participants during the 2nd-5th days of the menstrual cycle. Hormonal and metabolic variables of all participants were assessed. Insulin resistance was calculated using the homoeostasis model assessment formula (15). Body mass index (BMI) and Waist/Hip ratio (WHR) were determined.

Chemiluminescent microparticle immunoassays (Abbott, Architect i2000, Abbott Laboratories Diagnostics Division, IL, USA) were used to measure thyroid-stimulating hormone (TSH) and free thyroxine (fT_4_), free triiodothyronine (fT_3_). Chemiluminescent competitive immunoassays (Advia centaur XP, Siemens, Tarrytown, USA) were used to measure the anti-thyroglobulin antibody (anti-Tg Ab) and anti-thyroperoxidase antibody (anti-TPO Ab). The lower and upper limits were as follows: fT_3_: 2.5-3.9 pg/mL; fT_4_: 0.58-1.60 ng/dL; TSH: 0.38-5.33 μIU/mL; anti-Tg Ab: 0-60 IU/mL; anti-TPO Ab:0-57 IU/mL.

Thyroid USG was performed with a high-resolution ultrasound machine (Hitachi, Japan; EUB 7000) that had a 6-14 megahertz linear transducer. Only one operator performed all the measurements. Lesions over 3 mm (diameter) on USG were considered as nodules. The elliptical shape volume formula (0.479 x length x width x height) was used to calculate the volume of each thyroid lobe, and total thyroid volume (TV) was calculated by adding the right and left lobe volumes.

### Statistical analysis

The SPSS statistical software (version 17; SPSS, Chicago, IL, USA) was used to perform the statistical analysis. Fisher’s exact test or Chi-square test were used to analyze categorical variables. Normality of the variables was tested using the Kolmogorov-Smirnov test. The Mann-Whitney U test and independent samples t-test were to compare groups. Data are expressed as mean ± standard deviation or median with interquartile range as appropriate. Continuous variables were evaluated using Pearson’s correlation coefficient, and Spearman’s rho correlation coefficient test was used to evaluate non-normally distributed variables. P values less than 0.05 were considered statistically significant.

## RESULTS

The study included 97 patients with PCOS (mean age, 24.1±6.0 years) and 71 controls (mean age, 24.4±4.5 years). Table 1 represents the general characteristics of the patients and controls. There were no significant differences in terms of age and total cholesterol levels between the groups (p>.05). The BMI, WHR, triglyceride (TG), low density lipoprotein cholesterol, fasting blood glucose, fasting insulin and homeostasis model assessment insulin resistance (HOMA-IR) were significantly higher in patients with PCOS than in the control group (p<0.001, p<0.001, p=0.023, p<0.001, p<0.001, p<0.001, p<0.001, respectively). High density lipoprotein cholesterol was significantly higher in the control group (p=0.005).

The TSH and fT_4_ levels were similar in both groups. Table 2 represents the thyroid examination of the patients and controls. The mean TV was 11.4±4.7 mL in patients with PCOS and 9.9±2.8 mL in healthy controls (p=.022). In the PCOS group and controls, thyroid nodules were detected in 29/97 (29.9%) and 11/71 (15.5%), respectively (p=.043). The number of patients with PCOS who had a single nodule was 23 and multiple nodules was 6. In contrast, 6 control subjects had a single nodule and 5 had multiple nodules.

Patients with PCOS had higher a prevalence of positive anti-Tg Ab than controls (16.5% vs. 5.6%) (p=.051). The prevalence of subjects with positive anti-TPO Ab among the patient and control groups was 32.0% and 15.5%, respectively. It was significantly higher in patients with PCOS (p=.019). The frequency of AITD was significantly higher in patients with PCOS [PCOS, 39/97 (40.2%); and controls, 11/71 (15.5%); p=.001] when we consider either thyroid heterogeneity and/or positivity of autoantibodies as AITD.

**Figure 1:**
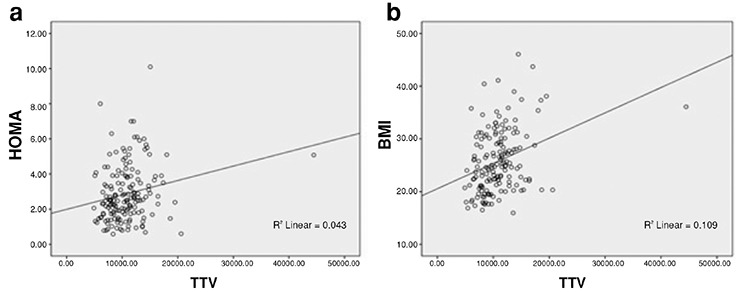
Thyroid volume was correlated with (a) homeostasis model assessment insulin resistance and (b) body mass index

There were statistically significant relationships between TV and age, BMI, fasting glucose, HOMA-IR, and fasting insulin (Table 3, Figure 1). The correlation between anti-TG Ab, anti-TPO Ab and TSH levels were also statistically significant (r=0.466, p<.001; r=0.218, p=.005, respectively).

The participants were divided into two groups according to the existence of thyroid nodules. Participants with thyroid nodules were older and had higher fasting glucose, fasting insulin, HOMA-IR, and BMI compared with those without thyroid nodules (Table 4). TSH, fT_4_, anti-Tg Ab, anti-TPO Ab, and total TV were similar both groups.

## DISCUSSION

Our study showed that nodule frequency and TV were significantly higher in patients with PCOS. A positive correlation was detected between TV and age, BMI, fasting glucose, fasting insulin, and HOMA-IR. Additionally, participants with thyroid nodules were older and had higher BMI, fasting glucose, fasting insulin, and HOMA-IR compared with those without thyroid nodules.

Duran et al. (16) investigated the nodular goiter prevalence in patients with PCOS. They reported that patients and controls had similar TV and nodule frequency. In their study, age and TV had a positive correlation. However, two different studies demonstrated that thyroid disorders had a high prevalence rate among young patients with PCOS compared with age-matched controls (17, 18).

TSH has important roles in the differentiation and growth of thyroid cells (19). In our study, serum TSH levels were normal and not different between patients and controls. In addition, TSH levels were similar between participants with- and without thyroid nodules. Therefore, TSH cannot be the only factor in the pathogenesis of nodule formation. Rezzonico et al. (12) reported the mitogenic effect of insulin on thyroid cell cultures. Other studies confirmed the relationship between thyroid nodule and insulin resistance (20). The reduction of thyroid nodule volume after amelioration of insulin resistance with metformin proved this causal relationship (21).

Patients with PCOS frequently have hyperinsulinemia and insulin resistance (14). We detected higher fasting insulin and HOMA-IR values in patients with thyroid nodules. The characteristic features of insulin resistance are hyperinsulinemia and impaired biologic response to insulin within target tissues (22, 23). It has been also shown that thyroid cancer cells have insulin receptors (24, 25). In light of our study results, we recommend USG evaluation for the presence of thyroid nodules in patients with PCOS with insulin resistance.

The association between AITD with PCOS is an another important topic. Kachuei et al. (26) and Janssen et al. (27) reported that the prevalence of autoimmune thyroiditis in PCOS was high (28, 29). We also detected statistically significantly high levels of anti-Tg and anti-TPO in patients with PCOS. The prevalence of positive anti-TPO Ab and anti-TgAb was higher in the PCOS group than in controls. Also the AITD’s frequency was significantly higher in patients with PCOS. Calvar et al. (28) demonstrated that young patients with PCOS had a high rate of AITD and this result was associated with high levels of fasting insulin and HOMA-IR. However, no correlation was established between autoantibody positivity and metabolic parameters such as BMI, insulin, and HOMA-IR.

In conclusion, we showed that insulin resistance was an important risk factor for increased TV and nodule formation. Patients with PCOS frequently have a thyroid disorder. Thyroid hormones are usually checked during the investigation of patients with PCOS; however, as a result of our study, serum thyroid autoantibodies and presence of thyroid nodule should also be investigated in these patients. The major limitation of our study is the determination of participants’ glucose status only by measuring fasting glucose and HOMA index. However, the Clinical Practice Guidelines of the Endocrine Society recommend using an oral glucose tolerance test (OGTT) to screen for impaired glucose tolerance and type 2 diabetes (29). Further studies should be designed with large numbers of participants using OGTT.

## Figures and Tables

**Table 1:**
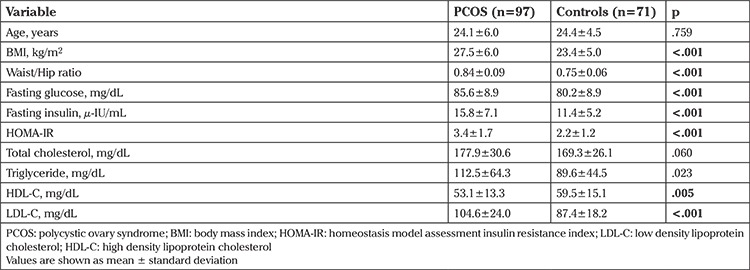
The clinical and biochemical data of patients with polycystic ovary syndrome and controls

**Table 2:**
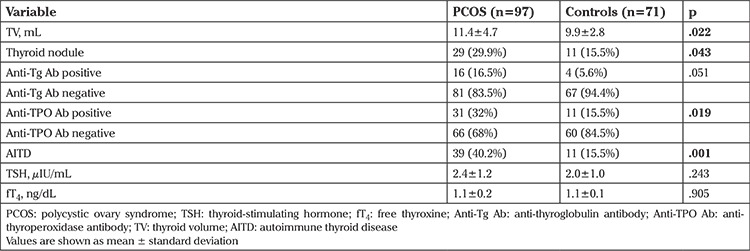
Thyroid test results of the patients with polycystic ovary syndrome and controls

**Table 3:**
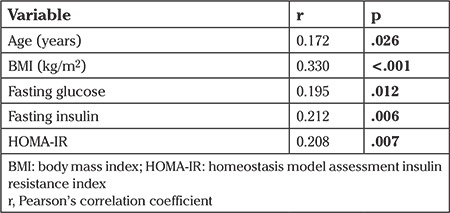
The correlation of thyroid volume with other parameters

**Table 4:**
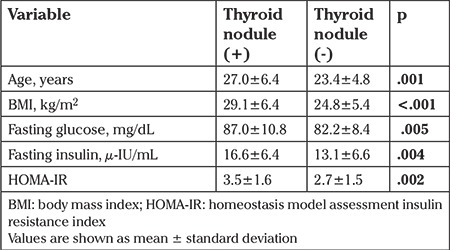
Comparison of clinical and biochemical data in participants with and without thyroid nodules
